# Supplementary material for: Association between epidemiological and clinico-pathological features of breast cancer with prognosis, family history, Ki-67 proliferation index and survival in Tunisian breast cancer patients
Source: PLoS One. 2022 Sep 12;17(9):e0269732. doi: 10.1371/journal.pone.0269732 (PMC9467370; doi:10.1371/journal.pone.0269732)
Supplement: S1 Table — (DOCX) [file pone.0269732.s001.docx]

**S1 Table. Epidemiological features of breast cancer patients**

| **Epidemiological features** | | | **Number of cases**  **N (%)** |
| --- | --- | --- | --- |
| **Family history of cancers** | **Familial/ hereditary breast cancer (N= 596)** | Yes | 139 (23.3) |
|  |  | No | 457 (76.7) |
|  | **Family history of breast cancer (N= 596)** | Yes | 167 (28) |
|  |  | No | 429 (72) |
|  | **Family history of ovarian cancer (N= 594)** | Yes | 12 (2) |
|  |  | No | 582 (98) |
|  | **Family history of other cancers (N= 571)** | Yes | 194 (33.98) |
|  |  | No | 377 (66.02) |
|  | **Personal history of cancers (N= 595)** | Yes | 12 (2) |
|  |  | No | 583 (98) |
|  | **Consanguinity (N= 182)** | Yes | 46 (25.3) |
|  |  | No | 136 (74.7) |
| **Reproductive factors** | **Oral contraception (N= 237)** | Yes | 171 (72.15) |
|  |  | No | 66 (27.85) |
|  | **Breastfeeding (N= 371)** | Yes | 287 (77.4) |
|  |  | No | 84 (22.6) |
|  | **Parity (N= 332)** | Parous | 316 (95.2) |
|  |  | Nulliparous | 16 (4.8) |
|  | **Breast cancer diagnosed during pregnancy (N= 579)** | Yes | 17 (2.9) |
|  |  | No | 562 (97.1) |
|  | **Menopausal status (N= 595)** | Premenopausal | 325 (54.6) |
|  |  | Postmenopausal | 270 (45.4) |
| **Other risk factors** | **Breast density (N= 109)** | BI-RADS1 | 18 (16.5) |
|  |  | BI-RADS2 | 30 (27.5) |
|  |  | BI-RADS3 | 44 (40.4) |
|  |  | BI-RADS4 | 13 (11.9) |
|  |  | BI-RADS5 | 4 (3.7) |
|  | **Comorbidities (N= 191)** | Cardiovascular diseases | 97 (50.79) |
|  |  | Diabetes | 35 (18.32) |
|  |  | Cardiovascular disease and diabetes | 6 (3.14) |
|  |  | Metabolic syndrome | 14 (7.33) |
|  |  | Rheumatologic diseases | 9 (4.71) |
|  |  | Others | 30 (15.71) |
