# Supplementary material for: Association between epidemiological and clinico-pathological features of breast cancer with prognosis, family history, Ki-67 proliferation index and survival in Tunisian breast cancer patients
Source: PLoS One. 2022 Sep 12;17(9):e0269732. doi: 10.1371/journal.pone.0269732 (PMC9467370; doi:10.1371/journal.pone.0269732)
Supplement: S2 Table — (DOCX) [file pone.0269732.s002.docx]

**S2 Table. Clinicopathological characteristics of breast cancer patients**

| **Clinicopathological Characteristics** | | **Number of cases N (%)** |
| --- | --- | --- |
| **Mean Tumor size** | 38.95 mm | **-** |
| **Molecular subtypes (N =601)** | Luminal A | 168 (27.95) |
|  | Luminal B | 278 (46.26) |
|  | HER2+ | 62 (10.32) |
|  | TNBC | 93 (15.47) |
| **Histological grade status (N=584)** | I | 62 (10.6) |
|  | II | 310 (53.1) |
|  | III | 212 (36.3) |
| **Histological type (N =602)** | IDC | 536 (89) |
|  | ILC | 16 (2.7) |
|  | MC | 22 (3.7) |
|  | Other | 28 (4.6) |
| **Intraductal component (N =452)** | Yes | 226 (50) |
|  | No | 226 (50) |
| **Multifocal tumors (N=509)** | Yes | 151 (29.7) |
|  | No | 358 (70.3) |
| **T stage (N =523)** | T0 | 6 (1.15) |
|  | T1-T2 | 398 (76.1) |
|  | T3 | 44 (8.41) |
|  | T4 | 75 (14.34) |
| **Inflammatory Breast Cancer (N =523)** | T4d | 28 (5.35) |
| **Lymph node involvement (N =511)** | Yes | 295 (57.7) |
|  | No | 216 (42.3) |
| **Ki-67 index status (N =509)** | Ki-67 ≤20% | 216 (42.44) |
|  | Ki-67>20% | 293 (57.56) |
| **Metastatic status (N =600)** | M0 | 513 (85.5) |
|  | M1 | 87 (14.5) |
| **Relapse (N =537)** | Yes | 132 (24.6) |
|  | No | 405 (75.4) |
| **Site of relapse (N =114)** | Bones | 28 (24.6) |
|  | Lung | 20 (17.5) |
|  | Liver | 9 (7.9) |
|  | Brain | 7 (6.1) |
