# Supplementary material for: Association between epidemiological and clinico-pathological features of breast cancer with prognosis, family history, Ki-67 proliferation index and survival in Tunisian breast cancer patients
Source: PLoS One. 2022 Sep 12;17(9):e0269732. doi: 10.1371/journal.pone.0269732 (PMC9467370; doi:10.1371/journal.pone.0269732)
Supplement: S1 File — (DOCX) [file pone.0269732.s003.docx]

**S1 File. Form model proposed for the investigation of epidemiological and genetics features of breast cancer in Tunisia**

**Patient informations**

1. First name: ………………… 2. Last name: ……………………………….

3. Medical record number:… …………………………………...................

4. Address: ………………………………………………………………………

5. Phone number ……………………………………………………………….

**Epidemiological factors**

1. Educational level ………………………………………………………..⬜

1. Primary 2. Secondary 3. University

2. Socio-economic level ……………………………………………………. ⬜

1. High 2. Middle 3. Low

3. Profession………………………………………………………………….

4. Civil status: …………………………………………...⬜

1. Married 2. Single

5. Place of residence: ………………………………………………….......⬜

1.Urban zone 2. Rural zone

6. Maternal geographical origin…………………………………………….

7. Paternal geographical origin…………………………………………….

8. Consanguinity ...……….……………………………...⬜

1. Yes 2. Endogamy 3. No

9. Mammographic density: .......................................................... ⬜

10.1. BIRADS classification :……………………………..

10.2 ACR classification :…………………………………..

10. Smoking: ................................................................⬜

1. No 2. Yes If yes, in which frequency………….

11. Alcoholism: ................................................................⬜

1. No 2. Yes If yes, in which frequency……………

12. Infertility: ................................................................⬜

1. Personal 2. Familial 3. No

**Genetic factors**

13. Personal history of cancer: ....................................... ⬜

1. No 2. Yes If yes, what type of cancer?...............................

Age at diagnosis

14. Personal history of rare genetic diseases..........................⬜

1. No 2. Yes ...If yes, which disease...................

15. Family history of breast cancer: ....................................... ⬜

1. No 2. Yes ...If yes, number of affected cases...................

1^st^, 2^nd^, 3rd of 4^th^ degree relatives................

Age at diagnosis

16. Family history of ovarian cancer: ....................................... ⬜

1. No 2. Yes ...If yes, number of affected cases...................

1^st^, 2^nd^, 3rd of 4^th^ degree relatives…………….

Age at diagnosis

17. Family history of other cancers: ............................................⬜

1. No 2. Yes ...If yes, what type of cancer?........................

number of affected cases...................

1^st^, 2^nd^, 3rd of 4^th^ degree relatives…………….

18. *BRCA1/2* genetic testing : 1. Negative 2. Positive 3. Not yet realized..........................⬜
